# Data sets for author name disambiguation: an empirical analysis and a new resource

**DOI:** 10.1007/s11192-017-2363-5

**Published:** 2017-03-27

**Authors:** Mark-Christoph Müller, Florian Reitz, Nicolas Roy

**Affiliations:** 1 Heidelberg Institute for Theoretical Studies, Heidelberg, Germany; 2 DBLP, Trier, Germany; 3 Mathematics Department, FIZ Karlsruhe, Berlin, Germany

**Keywords:** Author name disambiguation, Author name homography, Author name variability, Data sets, Digital libraries

## Abstract

Data sets of publication meta data with manually disambiguated author names play an important role in current author name disambiguation (AND) research. We review the most important data sets used so far, and compare their respective advantages and shortcomings. From the results of this review, we derive a set of general requirements to future AND data sets. These include both trivial requirements, like absence of errors and preservation of author order, and more substantial ones, like full disambiguation and adequate representation of publications with a small number of authors and highly variable author names. On the basis of these requirements, we create and make publicly available a new AND data set, SCAD-zbMATH. Both the quantitative analysis of this data set and the results of our initial AND experiments with a naive baseline algorithm show the SCAD-zbMATH data set to be considerably different from existing ones. We consider it a useful new resource that will challenge the state of the art in AND and benefit the AND research community.

## Introduction

In this paper, we provide a comprehensive and detailed review of data sets used in computational author name disambiguation (AND) experiments.^1^ AND data sets are basically collections of publication headers in which author names have been annotated with unique author identifiers. They are essential and indispensable resources for current research in computational AND, which is characterized by empirical, evaluation-based approaches (Ferreira et al. 2012a). AND data sets are utilized in two ways: First, computational AND approaches based on supervised machine-learning require them as training data during the development (Han et al. 2004; Treeratpituk and Giles 2009) or the parameter estimation phase (Santana et al. 2015). Second, they are also indispensable as test or reference data (often called *gold standard* or *ground truth* data) in AND system evaluation. Here the disambiguation decisions of the system under evaluation are compared to the correct disambiguations encoded manually in the data set, and the system performance is quantified on the basis of the number of correct and incorrect decisions. When a new AND data set is created as part of a research project, the design of this data set will probably reflect some explicit or implicit assumptions about the task. Fan et al. is an example of a project where the main research interest is in co-author networks, and where an algorithm is presented that uses author information only (Fan et al. 2011). Accordingly, Fan et al. create a data set of approx. 760.000 publications which contains *only* author names. What is even more important, Fan et al. systematically exclude from their data set all publications with only one author, because these are not accessible to co-author-based approaches. In contrast, Song et al. focus on disambiguating authors by using advanced semantic topic-modelling techniques (Song et al. 2007). They create a data set of more than 750.000 publications which contains author names, but also titles, abstracts, keywords, and the full text of each publication’s first page.

When the creation of a new data set is out of the scope of a project, the choice of existing data sets available for re-use will have an effect in terms of applicable methods, and, ultimately, outcomes. To give an example, as we will show in the section “Data set content analysis”, most data sets annotate only one, rarely two authors per publication with unique author identifiers, while the other authors remain unidentified and thus undisambiguated. For these authors, co-author network analysis, which is a cornerstone of many AND algorithms, simply has to assume that superficial, string-based name identity always implies identity of the author individual. Likewise, superficially *different* names will be treated as referring to different author individuals. Both assumptions, however, are obviously not valid. Co-author ambiguity, e.g., is present if the names of one or more unidentified authors in a publication are also used by different authors in other publications. It causes a disambiguation algorithm to incorrectly lump together these authors on the basis of matching names, thus producing incorrect connections in the resulting co-author network. In their discussion of open challenges for AND, Ferreira et al. explicitly refer to cases of co-author ambiguity as *very ambiguous cases*, also pointing out that the problem might be more pronounced for Asian names (Ferreira et al. 2012a). Shin et al. re-use data sets originally created by Han et al. (2005a, b) and Wang et al. (2011), where authors are also only partly identified (Shin et al. 2014). And in fact, in the error analysis of their co-author-based system, Shin et al. identify co-author ambiguity as one of three major sources of error. These examples show that there is a strong mutual interaction and dependency between current research in computational AND, and the AND data sets used in this research.

Our point of departure in this paper is the following: Current state-of-the-art AND systems like *Nearest Cluster* (Santana et al. 2015) or *BatchAD+IncAD* (Qian et al. 2015) report very good performance on distinct, but comparable, data sets: Santana et al., for their solely batch-based system, report a *K* score of 0.940 on the KISTI data set, and a *K* score of 0.917 on the BDBComp data set.^2^ Likewise, the system of Qian et al., who use the $\hbox {B}^3$ evaluation measure (Bagga and Baldwin 1998), yields a F1 score of 86.83 when run in batch-mode (BatchAD) on a similar, DBLP-derived data set. While these results as impressive, there are aspects to real-life AND which are simply not well-represented in the respective data sets. Potential co-author ambiguity has already been mentioned above. Other aspects include

1. cases where one author appears under several names with *non-trivial differences* (as opposed to differences that are only due to abbreviated first or middle names),
2. cases where the actual author name is written in a non-western (e.g., Asian or Cyrillic) alphabet and appears in the publication header in some transliterated version, which in turn can give rise to instances of case 1,
3. cases of publications by less productive authors or authors with only a small number of collaborators, for which rich co-author information is not available, and
4. cases of publications from scientific fields or communities that generally tend to have smaller numbers of co-authors.

The aim of this paper is two-fold: First, by means of an analysis of the most prominent data sets used in AND research so far, we want to identify and suggest new directions for research in AND. Second, we want to facilitate AND research by designing, creating, and making available a novel AND data set which complements existing ones. We do this by utilizing data and expertise available at the two major bibliographic data bases DBLP^3^ and zbMATH.^4^

The rest of the paper is structured as follows: In the section “Background”, we briefly outline some key concepts of AND and provide some definitions that will be used throughout the paper. The section “Review of AND data sets” contains a detailed review of the most important AND data sets. To our knowledge, this is the first comprehensive overview of this kind. In the section “A new AND data set from the domain of mathematics”, we provide some background information on the real-life data that we employ for the creation of our own data set, **SCAD-zbMATH**,^5^ and describe the quality assurance process. The paper ends with conclusions and an outlook in the section “Conclusion”.

## Background

In author name disambiguation (AND), *publication* and *author* are two central concepts. The authors of a publication are denoted by their names, for which, in case of multi-author publications, the list position in the order of appearance in the publication header may also be relevant.

Each tuple of author name, author name position in author list, and unique publication identifier^6^ constitutes an *authorship record* (Cota et al. 2010). Using this terminology, author name disambiguation can then be characterized as follows: Given a set of authorship records, AND tries to determine which of these refer to the same author entity. This task is very similar to the co-reference resolution task in Natural Language Processing (NLP), which tries to identify all expressions in a document that refer to (or *mention*) the same entity (Ng 2010).^7^ AND is made difficult by two characteristics of person names:

Distinct individuals bear, and publish under, the same name, which gives rise to authorship records with matching author names, but distinct underlying author entities. This phenomenon is called *name homography*.^8^ Failure to distinguish between different authors with identical names will cause a merging or *Mixed Citation* (Lee et al. 2005) error.

Likewise, different names can be used to refer to the same author entity, which produces authorship records with different author names, but relations to the same author entity. This phenomenon is known as *name variability*.^9^ Failure to correctly merge these records will result in fragmentation (Esperidião et al. 2014) or *Split Citation* (Lee et al. 2005) errors. Note that, strictly speaking, the term *disambiguation* applies to cases of name homography only.

Due to the existence of name homography and name variability, author names as they appear in publication headers or publication meta-data are often not sufficient to uniquely identify and distinguish between authors.

It is worth noting that author name ambiguity which results from *variability* is, at least in part, a home-made problem: While name *homography* will always exist as the result of a limitation of available person names, name *variability* is sometimes simply due to a lack of consistency on the part of authors and publishers. Authors sometimes deliberately use different variants, including abbreviations, of their first and middle names, while publishers often abbreviate first names to initials. For example, of all signatures which were added to DBLP between 2011 and 2015, 12.8% were delivered with **all** first name components abbreviated. McKay et al. point out that some authors use name variations to separate different areas of research or to hide their gender. They also report that researchers might change their name on a publication to avoid confusion with authors who have a similar name (McKay et al. 2010). Already in 1995, Grossman and Ion identified this lack of consistency as a central problem for citation studies in the field of Mathematics, and made a plea to authors to use their complete names consistently for each publication (Grossman and Ion 1995). The *degree* of author name ambiguity, however, seems to be different in different languages. In some Asian languages, e.g., the name homography problem is very pronounced: In the Chinese language area, it is estimated that the top three surnames (“Wang”, “Zhang”, and “Li”) account for about 21% of the population (Jin-Zhong et al. 2011). In the Vietnamese language area, a mere one hundred family names are presumed to be in common use,^10^ with the last name “Nguyen” accounting for up to 46% of family names.^11^ These are examples of name homography arising from cultural or ethnological conditions in the respective language areas. On the other hand, name variability can be very pronounced for languages using a non-western alphabet where the author names have to be transliterated into standard characters in order to facilitate search using a standard international keyboard. This is true again for Asian languages, but also for those using a Cyrillic alphabet, or an alphabet with special characters or diacritics. For all these, there are often several ways in which a name can be represented. Consider the following name variants actually observed in zbMATH:

**(Henryk) Żoł** ${\boldsymbol {\c{a}}}$ **dek** 12: Żoł${\c{a}}$dek; Żołądek; Zoł${\c{a}}$dek; Ẓoł${\c{a}}$dek; Żolądek

**Mefodij F. Raţă** 13: Raţă, Mefodij F.; Rata, Mefodie; Ratsa, Metodie; Raţă, Metodie

**(Ivan D.) Pukal’s’kyĭ** 14: Pukal’s’kyĭ; Pukal’s’kij; Pukal’skii; Pukal’skij; Pukal’s’kyj; Pukal’s’kyi; Pukal’skyj; Pukals’kyj; Pukal’skyj; Pukal’skiĭ; Pukal’sky; Pukalskyi; Pukalskyj; Pukalsky;

Author name ambiguity poses a major problem for online bibliographic data bases, which typically organize and make accessible publication data on the basis of author names. In order to be able to perform an author-targeted query, i.e., to retrieve all and only those publications by a particular author, the authorship records for this author need to be disambiguated. This type of query has been shown to be predominant in the navigation patterns of users searching for scholarly material.^15^ Without disambiguation of the bibliographic data base, precision and recall of this type of query are not guaranteed to be satisfactory (Salo 2009). But users of bibliographic data bases are not just researchers looking for other researchers: Scientific organizations and policy makers often rely on author-based statistics as a basis for critical action, while universities and research agencies often use publication statistics for their hiring and funding decisions. Weingart discusses the importance of bibliometrics for grant acquisition and the filling of positions (Weingart 2005). Frey and Rost, and the work referenced there, discuss the effects of publication-based ranking on scientific careers (Frey and Rost 2010). McKay et al. state that building a clean citation profile is a concern of many researchers (McKay et al. 2010). Finally, Diesner, Evans, and Kim, and Kim and Diesner, coming from a slightly different angle, provide evidence that naive, incorrect identification of authors based on name identity alone can have a distorting effect on scientometric analyses of both individual authors and entire scientific sectors, rendering the results of these analyses unreliable (Diesner et al. 2015; Kim and Diesner 2016).

All this makes author name ambiguity a relevant practical problem with far-reaching effects even outside the scholarly domain. As a consequence, online bibliographical data bases expend a lot of effort on author name disambiguation (cf. sections “Data curation at DBLP” and “Data curation at zbMATH”) in order to keep up a high quality of their author data, which is often stored in the form of disambiguated author profiles (Ley and Reuther 2006; Ley 2009). These efforts also include attempts to involve the author or user community (Mihaljevic-Brandt et al. 2014). The ever-growing number of scientific publications makes the task more and more difficult. Bornmann and Mutz, for example, report an exponential growth of publications by year for the period 1980-2012 (Bornmann and Mutz 2015). This tendency calls for automated methods, which in turn require data sets for their development and evaluation.

## Review of AND data sets

The following review is based on our survey of the current research literature in computational AND. To our knowledge, it is the first review of its kind. Ferreira et al., in their survey of AND systems, only briefly mention some data sets, but do not give any details (Ferreira et al. 2012a). Kang et al. and Ferreira et al. provide more, and more detailed, information in their respective sections on related work, including some statistics (Kang et al. 2011; Ferreira et al. 2012b). We, in contrast, performed a comprehensive analysis of current, empirically-oriented publications on AND and identified what we think are the most important data sets. In order to be included in our review, the data sets had to be sufficiently identifiable. This was the case for all data sets that were either made publicly available by the respective authors, or that we could obtain otherwise. Another requirement was that the data sets had to be freely available for non-commercial purposes, without additional restrictions or obligations for the individual re-using the data.

It will become clear in the following that all data sets obtained this way cover the domain of computer science, and that most of them are somehow based on DBLP data. This, however, is not a bias of our selection, but it reflects a reality of the AND research community: While there are several AND projects for other domains (most notably the Author-ity project^16^ for MEDLINE and PubMed), the data sets produced in these projects failed to reach a level of re-use in current projects that would have made them eligible for our review.^17^

In the following review, descriptive categories applied to each data set include the following:

- Does the data set contain errors or structural ambiguities, and what is its overall quality?
- Is the data set fully or only partially disambiguated? This property relates to the question whether or not *all* authorship records in the data set have a unique author identifier.
- Are the author names given in their full or only in abbreviated form?
- Is the author ordering of the original publication retained in the data set?
- Was the data set created in a methodologically controlled manner?
- Is the data set expandable by means of an external link to some other data source?

In order to facilitate analysis and comparison of the available data sets, we converted them from their various technical formats into a **canonical XML representation**. The main feature of this representation is that it puts the **publication** (and not the **author**) at its center. The author-centric perspective singles out one particular, *featured* author of a publication^18^ by annotating it with a unique author identifier. In doing so, the featured author’s co-authors in this publication are reduced to mere string-valued attributes of the featured author’s authorship record. In a fully publication-centric data set, on the other hand, **every** author of **every** publication is uniquely identified. This is a prerequisite for the development and, in particular, the accurate evaluation of co-author-based disambiguation methods: In a publication-centric data set, co-author relations are no longer established on the basis of string-matching, but on the basis of previous decisions made by a disambiguation method which is equipped to correctly handle co-author ambiguity and variability. In other words: Publication-centric data sets give up the fixed distinction between *featured* author and *co*-author in favor of a dynamic setup in which every author in turn is disambiguated. This also better reflects the situation in a realistic, production AND setting, where *every* author (and not just those with highly ambiguous names) will eventually be subjected to disambiguation.^19^

It is important to distinguish the merely **formal**, technical aspect of the data set from its **content**: Converting a data set into our canonical representation alone does not render it publication-centric, unless complete author identifiers are added. Likewise, however, an author-centric data set which happens to contain disambiguated authorship records for more than one author of a publication is (at least to some degree) publication-centric.

**Fig. 1:**
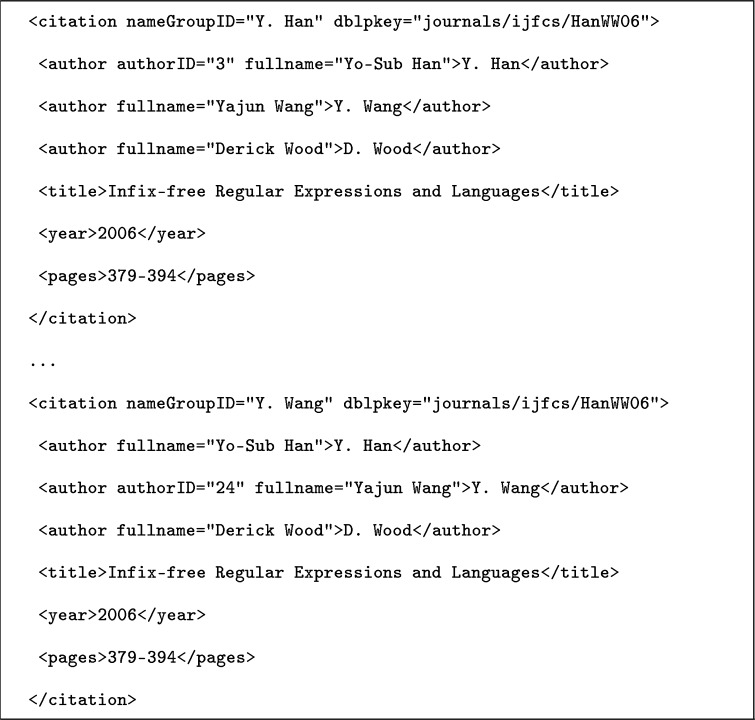
Author-centric information in the **KISTI-AD-E-01** data set

Consider Fig. 1, which shows an excerpt from the original XML version of the **KISTI-AD-E-01** data set. We chose this example because of its clarity; similar observations can be made in many of the other data sets.

**Fig. 2:**
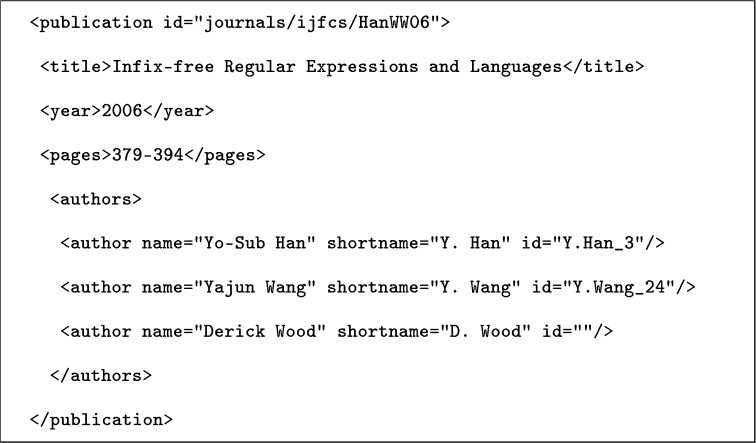
Information from Fig. 1 in canonical, publication-centric format

The example shows two citation entries from different parts of the XML file. Each entry corresponds to one authorship record, which renders this data set author-centric. The dblpkey attribute is the publication identifier, and entries with the same value represent the same publication. Note that each citation entry also has a nameGroupID attribute, which groups entries belonging to one so-called ***same-name group***.^20^ We found that these groups appear (in one form or another) in many of the reviewed data sets, and they often (e.g., in the **Han-DBLP**, **REXA-AND**, and **Wang-Arnetminer** data sets) are the major means of data set organization. In most cases, a same-name-group is identified by a first-name initial and a full last name (Y. Han and Y. Wang in the example). In each citation entry in Fig. 1, a different author is disambiguated by means of an author identifier. Technically converting an author-centric data set like this into a publication-centric one involves merging individual authorship records on a common value in the publication identifier. An excerpt from the result of this conversion is shown in Fig. 2.

Note that in the example no author identifier has been added to the third author, as this manual disambiguation would constitute a non-trivial enhancement of the data set, which is outside the scope of this review.

### Data set content analysis

This section describes six AND data sets, whose main properties are provided in Table 18 in the appendix. The **Han-DBLP** 21 data set is one of the first and most influential data sets in AND. It was originally created and employed by Han et al. (2005a, b), with a previous version described in Han et al. (2004). As the name suggests, **Han-DBLP** is based on data from DBLP, which was obtained using the publicly available download function. The inclusion of authorship records into the data set was based on the degree of ambiguity of the respective author name. This was determined by clustering all author names according to their first name initial and full last name, and ranking the clusters according to their in-cluster name variability, i.e., according to the number of distinct full author names, such that highly ambiguous combinations of first-name initial and last name ranked top. The top four clusters determined in this way were “J. Lee”, “S. Lee”, “Y. Chen”, and “C. Chen”. These were complemented by ten other highly ambiguous clusters, resulting in a total of 14 clusters. The statistics of the original data set can be found in Han et al. 2005 (Han et al. 2005a, b). Han et al. 2005 contains more detailed statistics, including a break-down of the individual name clusters (Han et al. 2005b, p. 338). For this data set, and for all other data sets in this review, unless otherwise noted, our own statistics were calculated on the basis of our converted version of the publicly available^22^ data set. Due to structural ambiguities in the original data set, automatic identification of the featured author was not possible for some records.^23^ This is the reason why the number of records with ID for **Han-DBLP** in Table 18 is actually lower than the total number of publications. Table 1 shows a sample of an authorship record from the “DJohnson” block of the **Han-DBLP** data set. In this case, the name of the featured author is given in short form only, while the names of the co-authors are given in the original form, which in most cases is more complete. Other records in this data set provide the complete names for all authors. The original publication ordering of the authors is not retained in the data. This sample record is typical of the **Han-DBLP** data set, as it contains one featured author only.

**Table 1:** Han-DBLP sample record

Pub-ID	–			
Title	Information-Theoretic Analysis of Neural Coding			
Venue	Journal of Computational Neuroscience			
Year	–			
Author-Pos.	?	?	?	?
Original name	–	Chandran Seshagiri	Charlotte M Gruner	Keith A Baggerly
Short name	D Johnson	–	–	–
Block	D Johnson	–	–	–
Author-ID	8	–	–	–

Inspection of the raw data set also showed that there was some degree of duplication, where identical data records appeared more than once, but in slightly varying form. However, no merging was possible due to the lack of publication identifiers.

No information is available about the methodology of data set creation, e.g., number of human annotators or quality assurance measures. Han et al. only mention a couple of heuristics based on, e.g., email and affiliation matches, and co-author name matches. It is unclear to what extent existing disambiguation information from DBLP was used. A recent analysis of the **Han-DBLP** data set revealed that it contains other serious errors, some of which appear to result from accepting incorrect author assignments from DBLP without verification (Shin et al. 2014). Santana et al. report similar findings (Santana et al. 2015).

Culotta et al. create the **Culotta-REXA** data set (Culotta et al. 2007). The basic statistics in Table 18 cover the full, publicly available^24^ data set of 13 name clusters, while Culotta et al. apparently use the top 8 clusters only. The slight mismatch between the number of authorship records with ID (3.015) and the total number of publications (3.007) indicates that there is a small number of publications with more than one disambiguated author, which could be merged on the basis of (internal) publication identifiers in the data set. Table 2 contains a sample record from the “jordan_m” block of the **Culotta-REXA** data set. In this data set, the author ordering of the original publication is preserved. In the sample record, the featured author, who is explicitly identified in the original data, and who can occur at any position in the author list, is only represented with the first name reduced to an initial, but other cases do also exist. The raw data set also contains some email addresses and affiliation information. Since they were often either incomplete or redundant, mapping them to the correct authors was not possible, so they were dropped during conversion. In addition, there are abstracts for 747 (25%) of the publications, which were retained in the converted data set.

**Table 2:** Culotta-REXA sample record

Pub-ID	–			
Title	Accurate Building Structure Recovery from Aerial Imagery			
Venue	–			
Year	–			
Author-Pos.	1	2	3	
Original name	Cocquerez, Jean Pierre	Cord, Mathieu	–	
Short name	–	–	Jordan, M	
Block	–	–	jordan_m	
Author-ID	–	–	MichelJordan	

In the context of their work on person name disambiguation, Wang et al. create three data sets, one of which is relevant for AND (Wang et al. 2011). The **Wang-Arnetminer** data set contains authorship records that were extracted from data collected within the Arnetminer system. Major sources for these data are DBLP, IEEE, and ACM. The basic statistics in Table 18 are based on our conversion of the simple^25^ version of the publicly available^26^ data set. The difference of 73 between the total number of publications and the number of authorship records with ID corresponds to the same number of publications with two disambiguated authors each. Merging of the distinct, author-centric records from the original data set was possible because it contains a consistent, though only internal, publication identifier. Table 3 provides a sample authorship record from the “R. Ramesh” block of the data set. Here, author ordering is again preserved, as well as case information.

**Table 3:** Wang-Arnetminer sample record

Pub-ID	738300			
Title	A dynamic learning model for on-line quality control using the TAGUCHI approach			
Venue	Applied Artificial Intelligence			
Year	1992			
Author-Pos.	1	2	3	
Original name	–	–	Ram Ramesh	
Short name	H. Raghav Rao	M. V. Thirumurthy	–	
Block	–	–	R. Ramesh	
Author-ID	–	–	5	

The annotation was performed in a methodologically controlled way, with each publication being annotated by at least three annotators. In total, 22 annotators were involved in the process. Accessible knowledge sources for the annotation included the respective authors’ home pages and the affiliation and email addresses in the publications. Wang et al. do not give any information on the frequency and type of observed cases of disagreement, but state that they were resolved by majority voting (Wang et al. 2011).

Kang et al. describe the creation of the **KISTI-AD-E-01** 27 data set (Kang et al. 2011). Unlike most other examples in this review, this data set is not a by-product of an AND research project, but the main focus of the work. The authors, again, used DBLP as the data source and started by collecting the 1.000 most frequent author names, where the frequency was computed by grouping and counting all author names appearing in approx. 870.000 publication records. Then, for each of the (potentially ambiguous) names, the complete publication list was retrieved from DBLP. On the basis of this, the actual disambiguation was performed by an elaborate, semi-automatic process involving web queries employing some of Google’s advanced search features. Manual inspection of the search results included, among other things, separating actual author home pages from digital library pages (like DBLP), and merging cases where authors maintained several home pages at, e.g., different institutions. Kang et al. report that cases which could not be disambiguated with reasonable manual effort were dropped. According to Kang et al., the original data set should be publicly available on the web site,^28^ but a download link was not available at the time of this writing. The copy of the original **KISTI-AD-E-01** data set used in this review was obtained from Alan Filipe Santana. The basic statistics in Table 18 show a considerable difference of 4.061 between the total number of publications and the number of authorship records with ID: As already mentioned above, the **KISTI-AD-E-01** data set contains many publications with two or more featured authors. Merging the separate authorship records was possible because they contained DBLP publication identifiers, which are not only internally consistent and unique, but which can also be used to recover additional publication meta data from DBLP. Table 4 contains a sample record from the “A. Datta” block. Note that this data set also contains systematically abbreviated names for all authors, along with the original, full names, where available.

**Table 4:** KISTI-AD-E-01 sample record

Pub-ID	conf/pomc/AnceaumeDGS02			
Title	Publish/subscribe scheme for mobile networks			
Venue	–			
Year	2002			
Author-Pos.	1	2	3	4
Original name	Emmanuelle Anceaume	Ajoy Kumar Datta	Maria Gradinariu	Gwendal Simon
Short name	E. Anceaume	A. Datta	M. Gradinariu	G. Simon
Block	–	A. Datta		–
Author-ID	–	2	–	–

Cota et al., apart from using versions of Han-DBLP and KISTI-AD-E-01, also create their own data set, **Cota-BDBComp** (Cota et al. 2010). This data set is based on the Brazilian Digital Library of Computing and covers both English and Portuguese publications. Table 18 gives basic statistics of our converted version of the publicly available^29^ data set. Table 5 provides an example. There is exactly one featured author for every publication, and the entire authorship record is given in lower case. The ordering of the authors of the original publication is not retained either. This is one of the few data sets which do not contain pre-defined block information, but global author identifiers (here: “114”).

**Table 5:** Cota-BDBComp sample record

Pub-ID	–			
Title	Towards a web service for geographic and multidimensional processing			
Venue	vi simposio brasileiro de geoinformatica			
Year	–			
Author-Pos.	?	?	?	?
Original name	–	–	–	joel da silva
Short name	v times	r fidalgo	r barros	–
Block	–	–	–	–
Author-ID	–	–	–	114

The **Qian-DBLP** data set, created and used by Qian et al. as test data, is the result of an aggregation of several existing data sets, including (versions of) Han-DBLP and Wang-Arnetminer (Qian et al. 2015). Table 18 gives statistics of our converted version of the original data set, which is publicly available.^30^ The actual creation of the data set by Qian et al. included cleansing and consolidation of the aggregated original data sets. This was performed by ten individuals and reduced the data set to a size of 6.783 records. Our analysis of the converted data set revealed that it still contained some duplicate records, which reduces its actual size to 6.716 records. Table 6 provides an example. Again, for each publication, there is exactly one featured author. The original author order is not preserved. Co-authors are given in full but lower case versions. Like Cota-BDBComp, this data set does not contain pre-defined block information. The **Qian-DBLP** data set is special in that it contains keywords for 5.720 publications (85%) and abstracts for 3.097 publications (46%). The abstracts, though, are often incomplete and incorrectly tokenized.

**Table 6:** Qian-DBLP sample record

Pub-ID	2545			
Title	An Approach to Composing Web Services with Context Heterogeneity			
Venue	International Conference on Web Services			
Year	2009			
Author-Pos.	?	?	?	?
Original name	Hongwei Zhu	xitong li	stuart e. madnick	yushun fan
Short name	–	–	–	–
Block	–	–	–	–
Author-ID	295	–	–	

### Quantitative analysis and comparison

All measures described in the following section were computed on the basis of the converted data sets, and string comparisons are strict (e.g., with respect to differences in diacritics) but case-insensitive. In all tables, the upper value in each cell is the absolute count, and the lower is the rounded percentage. Percentages may not add up to 100 in all cases due to rounding. Maximum values per data set are given in bold.

The **Publications per Author** statistic (Table 7) is a basic measure for the diversity of a data set. It is calculated by clustering all author identifiers in the data set by the number of publications in which they occur, and counting the size for each cluster. To illustrate, the value in the first column of the Culotta-REXA data set in Table 7 means that 213 of the 324 distinct authors included in the data set (=66%) are represented by exactly one publication. The Han-DBLP data set is exceptional in several ways: First, it does not contain any authors with just one publication. It is also the only one for which the largest single class of authors (99, or 21%) is represented by three publications in the data set, while the maximum value of 175 (=37%) is at the extreme end.

In the remaining five data sets, the largest single class of authors is represented by only one publication. For Culotta-REXA, Wang-Arnetminer, and in particular Cota-BDBComp, this class constitutes more than half of the respective data set. This means that for these data sets, the majority of authors are singletons, which can be expected to have an impact on clustering-based disambiguation methods. Cota-BDBComp is an extreme case, with almost 3 out of 4 authors being singletons. This characteristic of the data set has already been pointed out by Cota et al. themselves (Cota et al. 2010), and the ensuing difficulty was observed by Santana et al. (2015).

**Table 7:** **Publications per Author**. Identified authors with *n* publications

# Publications	1	2	3	4	5	6	7	8	9	10	>10	$\varSigma$
Data set												
Han-DBLP		66	99	39	36	17	16	17	9	5	**175**	479
		14	21	8	8	4	3	4	2	1	**37**	100
Culotta-REXA	**213**	44	14	10	6	2	1	3	4	1	26	324
	**66**	14	4	3	2	1	0	1	1	0	8	100
Cota-BDBComp	**151**	35	5	3	1	1	2		1		6	205
	**74**	17	2	1	0	0	1		0		3	100
Qian-DBLP	**440**	175	119	90	60	48	39	24	26	11	168	1200
	**37**	15	10	8	5	4	3	2	2	1	14	100
Wang-Arnetminer	**675**	199	96	52	33	32	19	12	14	11	114	1257
	**54**	16	8	4	3	3	2	1	1	1	9	100
KISTI-AD-E-01	**2864**	1071	655	461	317	215	168	136	116	91	827	6921
	**41**	15	9	7	5	3	2	2	2	1	12	100

The **Authors per Publication** statistic (Table 8) quantifies the amount of co-author information available in each publication in the data set. It is calculated by clustering all publications in the data set by their number of authors (both identified and unidentified), and counting the size of each cluster. To illustrate, the value in the third column for Qian-DBLP in Table 8 means that 1.850 of the 6.716 publications in this data set (=66%) have three authors. The figures in Table 8 show that in all six reviewed data sets, the largest single class of publications constitutes approx. 30% of the entire data set, with the top value being at $n=2$ and $n=3$ in half of the cases. This means that the majority of all reviewed data sets has publications with at least two authors. The Culotta-REXA data set has the highest percentage (15%) of single-author publications. For the other data sets, the values range from 3% (Cota-BDBComp) to 9% (KISTI-AD-E-01).

**Table 8:** **Authors per Publications**. Publications with *n* authors

# Authors	1	2	3	4	5	6	7	8	9	10	>10	$\varSigma$
Data set												
Han-DBLP	675	2410	**2537**	1462	697	312	164	77	40	20	59	8453
	8	29	**30**	17	8	4	2	1	0	0	1	100
Culotta-REXA	445	**965**	729	466	189	102	54	22	13	13	36	3034
	15	**32**	24	15	6	3	2	1	0	0	1	100
Cota-BDBComp	11	**104**	95	75	37	25	5	7	1		1	361
	3	**29**	26	21	10	7	1	2	0		0	100
Qian-DBLP	306	1416	**1850**	1548	863	337	172	78	51	31	64	6716
	5	21	**28**	23	13	5	3	1	1	0	1	100
Wang-Arnetminer	467	1548	**1804**	1458	708	315	148	77	40	25	66	6656
	7	23	**27**	22	11	5	2	1	1	0	1	100
KISTI-AD-E-01	3349	**11,694**	11268	6304	2738	1117	482	238	124	91	208	37,613
	9	**31**	30	17	7	3	1	1	0	0	1	100

The next two measures quantify the difficulty of disambiguating each data set on the basis of the distribution of author names and unique author identifiers. The **Author Name Homography** measure (Table 9) is calculated by collecting all distinct author names used by all identified authors, and counting the number of distinct identified authors who use these names. To illustrate, the value in the eleventh column of the Wang-Arnetminer data set means that 34 of 121 author names in this data set (=28%) are used by more than ten authors.

The KISTI-AD-E-01 data set contains both the full, original and the short names, and we calculate the measure for both. The difference in the total values for full and short names (6.250 vs. 881) is due to the fact that the latter conflates all names where the complete last name and the first letter of the first name are identical.

**Table 9:** **Author Name Homography**. Names applying to *n* identified authors

# Authors	1	2	3	4	5	6	7	8	9	10	>10	$\varSigma$
Data set												
Han-DBLP	**135**	8								1	13	157
	**86**	5								0	8	100
Culotta-REXA	**249**	26	8	5	4		2		1		3	298
	**84**	9	3	2	1		1		0		1	100
Cota-BDBComp	**235**	8	1	1								245
	**96**	3	0	0								100
Qian-DBLP	**473**	93	37	20	18	9	3	2	3	4	10	672
	**70**	14	6	3	3	1	0	0	0	1	1	100
Wang-Arnetminer	24	12	7	10	5	7	6	6	7	3	**34**	121
	20	10	6	8	4	6	5	5	6	2	**28**	100
KISTI-AD-E-01 (original names)	**5325**	679	138	50	33	14	4	2	2	2	1	6250
	**85**	11	2	1	1	0	0	0	0	0	0	100
KISTI-AD-E-01 (short names)	106	104	82	80	79	70	63	45	44	31	**177**	881
	12	12	9	9	9	8	7	5	5	4	**20**	100

The table shows that in almost all data sets the majority of (original) author names is used by exactly one author, with values at about 85% for three data sets and 70% for one data set. Actual name homography is present in all cases with $n>1$, so these four data sets display rather low homography. At the extreme ends, KISTI-AD-E-01 (Short names) displays a high degree of author name homography^31^ ($100\%-12\%=88\%$), while author names in Cota-BDBComp are homographs in as little as $100\%-96\%=4\%$ of cases. This latter observation can be explained by the fact that the Cota-BDBComp data set contains a high proportion of unabbreviated Brazilian authors, whose names tend to be longer and thus more distinctive.^32^

The **Author Name Variability** measure (Table 10) is calculated by collecting all distinct identified authors and counting the number of distinct author names that they use. To illustrate, the value in the second column for the KISTI-AD-E-01 (Full names) data set in Table 10 means that 556 of 6.921 identified authors (=8%) appear under two different name variants. Table 10 shows that for each data set in the review, the vast majority of authors uses exactly one name. Actual author name variability is present for all cases for $n > 1$, so there is hardly any author name variability. For the Wang-Arnetminer data set, the degree of name variability is below 1%, and for the Qian-DBLP data set, it is below 5%, while it is second-highest for the Han-DBLP data set with approx. 23%. KISTI-AD-E-01 (Short names) shows an extreme value of 100%. It is interesting to note that the 9% author name variability in KISTI-AD-E-01 (Full names) is exclusively due to variations in the *first* names, which is completely eliminated when they are reduced to initials.

**Table 10:** **Author Name Variability**. Identified authors appearing with *n* names

# Names	1	2	3	4	5	6	7	8	9	10	>10	$\varSigma$
Data set												
Han-DBLP	**370**	82	19	6	1	1						479
	**77**	17	4	1	0	0						100
Culotta-REXA	**273**	25	12	4	3	2	2		1		2	324
	**84**	8	4	1	1	1	1		0		1	100
Cota-BDBComp	**168**	29	4	2	1		1					205
	**82**	14	2	1	0		0					100
Qian-DBLP	**1153**	46	1									1200
	**96**	4	0									100
Wang-Arnetminer	**1245**	12										1257
	**99**	1										100
KISTI-AD-E-01 (original names)	**6292**	556	58	13	2							6921
	**91**	8	1	0	0							100
KISTI-AD-E-01 (short names)	**6921**											6921
	**100**											100

### Discussion

The results of our review so far can be summarized as follows: First, as already mentioned in the “Introduction” section, all of the reviewed data sets are **author-centric, i.e., only partially disambiguated**. The vast majority of publications identifies only one, rarely two authors, mostly on the basis of the degree of ambiguity which the name (in the form of first name initial plus full last name) exhibits. All other names remain unidentified. We, in contrast, argue that the availability of a fully disambiguated, publication-centric data set is necessary for developing and evaluating algorithms for realistic AND settings, in which all author names receive equal attention, and in which accurate co-author networks (based on *author* and not mere *name* identity) are required. Full disambiguation is indispensable as a diagnostic means for co-author-based systems, as it provides the only way to quantify co-author-based disambiguation errors and to estimate the potential benefit of perfect co-author information. However, in a fully disambiguated data set, there is no obvious way to distinguish between difficult and trivial disambiguation cases, because the mere existence of a unique identifier does no longer mean that the authorship record does actually pose a non-trivial disambiguation problem. Another, related consequence of full disambiguation is that the *overall* degree of homography and variability will be *lower* than in a partially disambiguated data set. This is because one challenging author in a given publication might be accompanied by one or more co-authors who are trivial to disambiguate, but who are included into the data set just the same. In the section “Initial naive baseline experimentation”, we describe a simple and practical procedure based on **selective disambiguation** which allows to maintain the advantages of full disambiguation, while at the same time focussing on relevant, ad-hoc sub sets of authorship records.

Second, we also found that in four out of six data sets, identified authorship records are **pre-assigned to hard-coded blocks**. The purpose of these blocks is to identify subsets of ambiguous authorship records, which correspond to same-name groups. These are then presented to the disambiguation system, which processes each group in turn and attempts to split it up into subgroups of uniquely identified authors. Often, performance measures of disambiguation algorithms using these data sets are also reported on a per-group basis (e.g., Santana et al. 2015). This way of representing and processing AND data completely ignores the fact that the creation of these groups, i.e., the *blocking*, is a non-trivial task in itself: In an actual bibliographic data base, prior to adding newly delivered publication records to existing (already disambiguated) ones, a pre-selection has to be done in order to select the candidates to which the new records could be linked.^33^ The performance and efficiency of the blocking method has a direct effect on the subsequent disambiguation: Low recall in blocking will definitely hurt recall in later disambiguation, if correct candidates are not considered, while low precision in blocking will increase execution time and potentially also hurt precision of disambiguation. Depending on the algorithmic complexity of the disambiguation system, an excessive number of candidates presented by the blocking method can render an otherwise effective disambiguation system unusable in a realistic setting with a non-trivial number of authorship records. However, given the way most of the reviewed data sets are structured and used, the potential of blocking errors is almost completely eliminated from AND.

Third, the qualitative analysis also showed that in many data sets **author first names were initialized**. In many cases (e.g., Han-DBLP and Culotta-REXA), this applied to the identified author only, while in other cases (e.g., Wang-Arnetminer and Cota-BDBComp), all *but* the identified authors were initialized. In all these cases, however, the abbreviation is *lossy* in the sense that the original name cannot be recovered, even though it might have been available in the original publication. This unnecessarily limits the usefulnes of the data sets, which, in order to be realistic, should contain the same amount of information as the publications that they are based on. While it is perfectly reasonable to systematically disregard information for evaluation purposes (e.g., by artificially initializing first names in order to create, or increase, ambiguity), this should be an *optional* limitation that should not be hard-coded in the data set. What is even more important, systematically disregarding information in a data set also leads to and encourages the development of methods that ignore the rich information that is, after all, contained in more complete, though still ambiguous, author names. In a realistic system, if more than the first name initial is available (which will often be the case), this information should be used, and applicable methods should be able to exploit it. The same is true for name structure: Many publications contain the author names in a *last name, first name* format, which might provide useful information for the development of e.g., blocking algorithms. Therefore, maximal author name information from the publication should be included, and internal name structure, if available, should be maintained. In addition, standard *abbreviated* names can also be supplied, such that methods using full and abbreviated names can be developed and evaluated in a systematic, reproducible manner. KISTI-AD-E-01 is an example of a data set that already contains both the more complete, original names, and short names, while Culotta-REXA is the only data set in our review that provides *structurally analysed* names that are systematically split into first, middle, and last name.

Fourth, our quantitative analysis found that the data sets contain only a **small number of single-author publications** (between 3 and 15%), with most publications having at least two or three authors. This distribution, however, is a property of the computer-science domain, and not universal, as in other domains, single-author publications are much more common. For these authors, established co-author-network based AND methods are not applicable. In order to support the development of methods for these cases, data sets with a stronger emphasis on single-author publications are required.

Fifth, the reviewed data sets show a clear **preference for author name** ***homography***, while author name *variability* is less pronounced. This is clearly visible in the way most data sets were created, and also in their resulting block-based structure, which reduces the AND task to separating distinct authors within the same block, rather than merging distinct author names under which one and the same author appears. Again, what is needed here is a new, complementary data set which treats author name variability as equally important.

Finally, among the more obvious and more easily met qualitative requirements for our data set are the following: Author ordering should be retained for the publications, as this is known to be a non-trivial aspect of publications (differently so in different domains), which is easy to represent in the data set, and which might turn out to be useful for AND or related tasks. We also argue for adding unique, externally valid publication ids to data sets, as these will allow a systematic access to additional publication data and meta-data, like e.g., key words, abstracts, or full texts. KISTI-AD-E-01 and Qian-DBLP already contain these types of links.

## A new AND data set from the domain of mathematics

In this section, we describe the new data set that we created in order to meet the requirements defined as the result of our review. In contrast to the other data sets in this review, the creation of our data set did not cause extra effort, because it took advantage of the fact that we had access to high-quality, manually curated data from DBLP and zbMATH. The section “Data bases: DBLP and zbMATH” describes some details about how data curation works at the two institutions, with a particular focus on semi-automatical versus purely manual curation and the different data quality levels resulting from this. The section “Quality assurance” provides details about the quality assurance measures that we employed to establish the validity of our data set, which is described in the section “The SCAD-zbMATH data set”.

### Data bases: DBLP and zbMATH

DBLP and zbMATH are two institutions which collect, curate, and make available bibliographic meta data. While they specialize in computer science and mathematics, respectively, there still is a significant overlap in the publications covered. Both institutions receive publication meta-data (e.g., journal and proceedings publications) from different publishers on a regular basis. While DBLP has already been the data source for many AND data sets, zbMATH data has not been used for this purpose so far.

#### Data curation at DBLP

The DBLP project collects and makes available meta data on publications in computer science and related fields. The project was established in 1993 by Michael Ley. Since 2011, DBLP has been a joint project between the University of Trier and the Leibniz-Zentrum für Informatik Schloss Dagstuhl, Germany. As of March 2016, the collection contains meta data of 3.3 million publications, which are linked to 1.7 million author profiles. Each day, more than 1.000 new publications are added.

The meta data for most publications is actively delivered by the publishers or by third parties such as conference organizers or journal editors. In other cases, meta data is obtained from the respective web sites by means of a set of specialized web crawlers. Regardless of the source of the data, a central requirement at DBLP is that all publications from a conference proceeding or a journal issue have to be added at the same time, because only this way it can be guaranteed that the data is both up-to-date and not unfairly balanced towards some author or authors (Ley and Reuther 2006).

For each incoming publication, the authors are automatically checked against the existing author profiles. In this process, the goal is to maintain **clean** author profiles, i.e., an authorship record should be assigned to an existing author profile only if this assignment can be done with high confidence. In order to find a matching author profile in DBLP, each author signature in a new publication is processed with several specialized string similarity functions (Ley and Reuther 2006). This processing step corresponds to **blocking**. Then, a simple social network analysis based on co-author information is performed to rank the potential candidate profiles. If an author profile is found, the authorship record is assigned, but only after the ranked candidate lists have been manually checked by a human data curator. In addition, if the matching author profile contains more details than the authorship record (e.g., the author’s full first name), the latter is expanded, and some other normalization is applied. This way, 10.6% of all new authorship records added between 2011 and 2015 had at least one abbreviated name part expanded during the initial data processing phase. In cases that remain unclear even after manual checking of candidates, in-depth checking, often involving external sources, is performed. However, the amount of new publications per day makes comprehensive detailed checking impossible, which inevitably leads to incorrect assignments. The described co-author-based candidate ranking works best for publications with many authors and for communities with a high average number of authors per publication. Thus, while the initial checking of author data ensures a basic level of data quality, a significant number of defective authorship records still find their way into the data base. Unless an authorship record has undergone explicit manual checking, its level of data quality will be expressed by the internally assigned status label *AUTO*.

To further improve data quality of existing authorship records, there is another, automated process which checks the whole data base on a daily basis. This process is able to detect errors in the data base that become evident only as a result of newly added data or corrected entries. It works by automatically analyzing the local co-author graph of each author for suspicious patterns. In general, a co-author graph is a representation of a co-author community, which is a group of authors who share at least one common publication. In a co-author graph, all directly collaborating authors are linked to each other. A **local** co-author graph for a specific author contains just this author and his or her direct collaborators. Given this input, the process can detect, for example, *distinct* author profiles that share a common co-author (Reuther 2006). This can be evidence for the profiles being incorrectly considered as cases of author name homography, when they actually are cases of author name variability. If, and only if, this is corroborated by a manual inspection, the data base is corrected by merging the author profiles. Name variability is a considerable problem for DBLP: In 2015, an average of 32.2 pairs of author profiles were merged each day.

For the detection of cases of author name homography, DBLP relies on manual work by data curators. Errors caused by author name homography are corrected by splitting the respective author profile. For author profiles that are suspected of lumping together several distinct authors with homographic names, an analysis is performed which also uses co-author community information. The analysis is based on a heuristic which assumes that distinct authors tend to have disparate, but internally connected, co-author communities, and it works by temporarily removing the author profile in question from its local co-author community. In the resulting graph(s), two co-authors are in the same community if they are still connected. If removal of an author profile yields two (or more) distinct co-author communities, this can be taken as evidence that the author profile in question actually lumps together several distinct authors, and that each of the remaining co-author communities belongs to a different author incorrectly merged in the profile. Using this heuristic, in 2015 an average of 5.3 author profiles were split each day.

Another important information source for homograph detection at DBLP is user or author contribution. For the period 2007–2010, 15.7% of all author profile splits were triggered by user emails (Reitz and Hoffmann 2011). Generally, corrections related to AND problems are more thoroughly checked than newly added data. We consider the data quality of publications which were involved in such changes as above average. Internally, the level of data quality of these cases is expressed by the status label *MANUAL*.

#### Data curation at zbMATH

zbMATH is the longest-running indexing and reviewing service in pure and applied mathematics. With currently more than 3.5 million entries with reviews or abstracts, zbMATH aims at fully covering the literature for the core areas of mathematics, while the area of “applications of mathematics”, such as natural sciences, computer science, economics, and engineering, is covered only partially. The zbMATH data base constantly increases its document collection, with a current annual growth of approx. 120.000 new entries and approx. 85 new journals, but it also contains historic documents.^34^ zbMATH covers several languages, and contains approx. 80% English, 5% Russian, 4% German, 4% French, and 3% Chinese publications. In most cases, titles of non-English publications are given both in their original language and in an English translation. Only about 1.8% of publications do not have an English title.

The editorial workflow at zbMATH is as follows. Each new document item that is submitted to zbMATH is examined by members of the editorial board, which is composed of several dozens of researchers with expertise in one or more relevant research areas. Documents considered relevant for zbMATH pass through an editorial process, in the course of which they are enriched with semantic meta data. This meta data comprises keywords, MSC codes,^35^ and possibly also a review written by an independent expert. No name normalization (except some LaTeX encoding of special characters and diacritics) is performed during the editorial process, which deals with document *content* only. Issues related to AND are addressed only afterwards. After the editorial process, the documents are added to the zbMATH collection.

The approx. 3.5 million documents in zbMATH correspond to about 6.1 million authorship records. The average number of authors per document is thus only about 1.7. This number is considerably smaller than in other areas, like experimental science or computer science, and illustrative of the authoring habits in the mathematical community. Authorship records in the zbMATH data base are associated with author profiles (currently approx. 930.000). See (Teschke 2009) and (Teschke and Wegner 2011). These author profiles are not only clusters of authorship records, but they also contain author-related meta-data like year of birth or year of PhD, derived meta data like publication span,^36^ or external identifiers that link them to author profiles in other services.^37^ What is special about the relation between authorship records and author profiles is that *a single* authorship record can be assigned to *several* author profiles. The assignment is done by a mixed manual-automatic **soft-clustering** process. If neither the system nor the zbMATH editors are able to choose a single author profile from several candidates, this process allows for some **ambiguity** in the authorship assignments. In this case, no new author profile will be created.^38^

In zbMATH, AND is performed as part of a more comprehensive author identification process. The process has both an **automatic** and a **manual** facet. As a matter of principle, results of manual processing have precedence over automatic decisions, and cannot be overridden or modified by the latter.

The automatic soft-clustering author disambiguation algorithm is **incremental**, **multi-featured**, and runs on a **daily** basis. The starting point of the algorithm at every day *D* is **1.** the whole collection of documents with their authorship records and **2.** autor profiles associated with authorship records as the result of the previous clustering on day $D-1$. At this step, in addition to the associated authorship records and the author-related meta data, each author profile also contains a temporal author profile, consisting of the publication span and information on potential gaps in the publication time line, and also information about all the different name and spelling variants used by the respective author. This latter information is automatically derived from the collection of authorship records.

The assignment of a given document is determined by successive filtering of a list of author profile candidates. This list is initially populated with profiles retrieved by a fast blocking algorithm which is based on exact string comparison of parts of the author name. Then the candidates are more finely analysed according to their full names (allowing e.g., for missing parts, abreviated names, and permutations), their temporal suitability (the document’s publication year is compared with the author’s temporal key data when available, like birth year, year PhD, etc.), and their co-author network (Jost et al. 2016).

After this analysis, the document is assigned to all author profiles in the candidate list. This gives rise to an average ambiguity of approx. 1.7 author profiles per authorship record.^39^ Note, however, that more than 97% of authorship records have an ambiguity of 1 (=no ambiguity) or 2. On the other hand, there are also authorship records with a very high ambiguity: At the time of this writing, the maximum is 912 for a publication authored by “Y. Wang”. If the algorithm cannot find any suitable candidate author profile for a given document, a new author profile is created. Data quality of the assignments made by this algorithm is expressed by the status labels *AUTO*_*UNIQUE* (for authorship records with ambiguity 1) and *AMBIGUOUS* (for authorship records with ambiguity ${>}1$).

Apart from this automatic disambiguation process, the zbMATH system also supports manual data curation. This can include the explicit confirmation of some earlier automatic assignment, its explicit removal, the creation of a new assignment, or some other modification. As mentioned above, all manual edits are *read-only* for the automatic disambiguation algorithm. The data quality of these cases is expressed by the status label *MANUAL*. The manual curation policy is author-centric, i.e., authorship records to be reviewed and edited are selected on a per-author basis. Manual edits often have a non-local character, in the sense that a modification on one autorship record assigment may have some effect on several other assignments, possibly even on those relating to publications by other authors. These side effects often emerge as a result of subsequent executions of the automatic disambiguation process. At the time of this writing, approx. 13.5% of the authorship records had been manually checked (status = *MANUAL*).

Finally, another important source of input for the manual data curation are the zbMATH users themselves. In summer 2014, zbMATH introduced a web-based interface dedicated to author disambiguation, which is accessible through an *Edit Profile* button at the top of each author profile on the zbMATH page (Mihaljevic-Brandt et al. 2014). This interface allows users to request^40^ edits not only of their own, but of any author profile. Supported edits include the correction of assignments of authorship records to author profiles, merging or splitting of author profiles, and providing additional meta data.

### Quality assurance

This section provides details about our experiment on a doubly annotated data set of authorship records from the DBLP and the zbMATH data bases. The aim was to quantify the correctness of the independent annotations of the two data sets, which is a requirement for using them as the basis for the creation of a gold standard data set. We quantified the correctness of the manual disambiguation in terms of the agreement between the two data sets, which is expressed as the $\hbox {B}^3$ score. As mentioned in the section “Data set content analysis”, similar quality assurance has been performed by some, but not all data sets in the review. Our experimental data set was created by intersecting individual data sets from DBLP and zbMATH. The process considered only manually assigned authorship records, since we wanted to compare the results of deliberate decisions made by experts, and not the performance of any automatic system or heuristic algorithm. An initial export produced one data set for DBLP (37.365 records) and one for zbMATH (47.289 records). We then created the actual intersection by mapping records from the two individual files on the basis of DOI and author position. The size of the intersection file was 2.886 authorship records in 2.779 publications. In order to quantify the disagreement, we used the CONLL scorer tool,^41^ which provides reference implementations for several evaluation measures, to compute the $\hbox {B}^3$ value between the DBLP and the zbMATH data set. Using DBLP as key and zbMATH as response,^42^ the results obtained were $R=99.70$, $P=99.32$, and $F1=99.51$. This indicated near-perfect agreement between the author disambiguation decisions. In other words: Given the same set of authorship records with homographic author names, the DBLP and zbMATH experts independently disambiguated the records in such a way that completely identical author clusters were formed in the vast majority of cases. We took this to be sufficient evidence for the actual correctness of the disambiguations.

For the cases of disagreement, however, we were interested in a more thorough, *qualitative* analysis.

For this, we used the scorer output to identify the publications containing the disagreements. This produced a list of 24 DOIs, which was then manually inspected by the data curation experts from DBLP and zbMATH. The result of this analysis was that all errors on the part of zbMATH were due to two reasons: Errors in the underlying data, which caused the author positions to be incorrect, and a few authorship records *incorrectly* labeled as *MANUAL* which were produced by a semi-automatic disambiguation component in the past. The remaining disagreements were all due to errors on the part of DBLP and, as was expected, consisted mostly in incorrect splits. All errors were corrected in the respective production data bases of DBLP and zbMATH, including the false *MANUAL* records that were involved in the observed disagreements.^43^

We concluded from this experiment that the level of quality in the zbMATH data base is sufficient for the data to be used as the basis of a new AND data set, without the need for additional manual annotation. Another result is that the quality of the same data in the DBLP data base is also very high. In particular, most errors in DBLP are incorrect *splits* (rather than *merges*), which is a manifestation of DBLP’s conservative, high-precision approach to author name disambiguation.

### The SCAD-zbMATH data set

For the creation of our own, novel data set, **SCAD-zbMATH**,^44^ we first created a full data set from the zbMATH production data base. Since we wanted our data set to be fully disambiguated, it was filtered to include only publications for which *all* authorship records were manually disambiguated. In general, meta data in zbMATH is not open data. Thus, while we wanted to create a useful data set of considerable size, at the same time we had to comply with several restrictions governing the use of the data. Accordingly, we had to remove all publications prior to the year 2000, along with all publications from particular journals or other venues. This left us with a complete data set of 271.663 authorship records. Due to additional licensing restrictions, only a subset of approximately 30.000 records could be used for the data set to be actually released. Thus, we had to drastically reduce the size of the data set, while at the same time maintaining, or even strengthening, its desirable properties. As described in the section “Discussion”, author name variability is one such property. In order to optimize the data set for author name variability, we selected all publications that contained at least one author who, in the full data set, appeared with six or more name variants. In order to also counter-balance the data set towards cases of name homography, we then added all publications that contained at least one author whose name was identical to the name of at least one other author. The filtering parameters were determined empirically, by creating temporary data sets and computing their author name variability and homography scores in the same way as described in the section “Quantitative analysis and comparison”. The threshold of six for author name variability might appear rather high, but it is justified because we wanted to aggressively filter the data set in order to reach the desired size of approximately 30.000 records. Also, as discussed in the section “Discussion” above, full disambiguation means that each actively selected author might be accompanied by one or more co-authors, who also increase the size of the data set. The statistics of the final data set can be found in the bottom part of Table 18 in the Appendix. Note also that the data set compares favourably to the six reviewed data sets with respect to the five qualitative properties. Table 11 provides a sample record. The distribution of languages in SCAD-zbMATH is slightly more skewed in favor of non-English publications, with 65% English, 12% Russian, 9% French, and 8% German publications. For all but approx. 2% of publications, however, an English title is provided.

**Table 11:** SCAD-zbMATH sample record

Pub-ID	zbmath:0738.35028			
Title	The nonlinear heat equation			
Venue	Proc. Math. Meet. in Honor of A. Dou, Madrid/Spain 1988, 251–258 (1989)			
Year	1989			
Author-Pos.	1			
Original name	Vázquez Suárez, Juan Luis			
Short name	Vázquez Suárez, J.			
Block	–			
Author-ID	vazquez.juan-luis			

As for the **Publications per Author** statistic (Table 12), it can be seen that 41% of the authors in the data set are represented by one publication only (either as a single author, or with co-authors). When compared to the other data sets (Table 7), this value is below average, and actually identical to that of KISTI-AD-E-01.

**Table 12:** **Publications per Author**. Identified authors with *n* publications

# Publications	1	2	3	4	5	6	7	8	9	10	>10	$\varSigma$
Data set												
SCAD-zbMATH	**1198**	414	223	166	110	76	68	46	41	32	572	2946
	**41**	14	8	6	4	3	2	2	1	1	19	100

Considering the **Authors per Publication** statistic (Table 13), the difference is much more pronounced, and also more relevant: SCAD-zbMATH publications are single-author publications in 83%. Also, the highest number of authors for a publication is seven, observed in only one publication, while all other data sets (Table 8) have at least one publication with more than ten authors. This is in line with our expectations, based on our experience with the publication habits in the field of mathematics. What is more, it is also a highly desirable property, as it will show the limitations of AND algorithms that heavily rely on co-author information.

**Table 13:** **Authors per Publications**. Publications with *n* authors

# Authors	1	2	3	4	5	6	7	8	9	10	${>}10$	$\varSigma$
Data set												
SCAD-zbMATH	**23,409**	4400	461	44	1	5	1					28,321
	**83**	16	2	0	0	0	0					100

With respect to the **Author Name Homography** statistic (Table 14), the SCAD-zbMATH data set shows a value of $100\% - 87\% = 13\%$ of original names being used by more than one author, and $100\% - 82\% = 18\%$ of short names. For original names, this value is again very similar to that of the KISTI-AD-E-01 data set.

**Table 14:** **Author Name Homography**. Names applying to *n* identified authors

# Authors	1	2	3	4	5	6	7	8	9	10	>10	$\varSigma$
Data set												
SCAD-zbMATH (original names)	**4091**	508	59	25	8	4		1				4696
	**87**	11	1	0	0	0		0				100
SCAD-zbMATH (short names)	**2380**	407	80	27	17	5	1		2			2919
	**82**	14	3	1	1	0	0		0			100

Finally, the **Author Name Variability** statistic of the SCAD-zbMATH data set (Table 15) is remarkable. The optimization of this data set property (by means of focussing on authors with many name variants) was one of the goals of the data set creation. The figures show that the data set indeed shows a very high author name variability: When the potentially longer, original names are considered, the author name variability is $100\% - 74\% = 26\%$, the highest value of all data sets when compared to Table 10. Note the peak of 158 at $n=6$, which is an effect of using this value as the name variability threshold for publication selection. Again, just like for KISTI-AD-E-01, the total number of original names is higher than the total number of short names, because in the latter variations in the first names are lost.

**Table 15:** **Author Name Variability**. Identified authors appearing with *n* names

# Names	1	2	3	4	5	6	7	8	9	10	>10	$\varSigma$
Data set												
SCAD-zbMATH (original names)	**2170**	383	61	10	7	158	64	39	25	13	16	2946
	**74**	13	2	0	0	5	2	1	0	0	0	100
SCAD-zbMATH (short names)	**2619**	138	83	47	31	15	7	1	3	1	1	2946
	**89**	5	3	2	1	0	0	0	0	0	0	100

In summary, while this is satisfactory, it would still have been nice if our efforts would have yielded a data set of more balanced difficulty. However, since we wanted to emphasize author name variability, we find this trade-off acceptable.

### Initial naive baseline experimentation

While the descriptive statistics provided above give some idea of the properties of a data set, it is also interesting to see how it behaves in an actual application situation, i.e., when it is subjected to some AND algorithm. The application of a full-blown, state-of-the-art algorithm is out of the scope of this paper, and will be part of future work. Instead, we chose to use a naive baseline algorithm which just treats author names as unique and unambiguous and clusters together authors with identical names. This approach is similar to the *all-initials* variant of the simple method described by Milojević, with the difference that our method optionally uses *all* available first name information, while the former uses *initialized* names only (Milojević 2013). Arehart and Miller, when evaluating name matching algorithms, use what they call the *Exact* algorithm as a similar baseline (Arehart and Miller 2008). Generally, the naive algorithm should yield better recall if the data set exhibits rather low author name variability, and better precision if it exhibits rather low author name homography. However, the interplay between data set variability, homography, and naive algorithm performance is more complex. In particular, comparably high author name variability does not automatically lead to poor recall. This is because the performance of the naive algorithm is also affected by the distribution of name variants among the authorship records of a particular author. To give just a simple example, imagine an author with six publications and two name variants. If the publications are split equally, i.e., 3–3 between the two name variants, the naive algorithm will produce two clusters of size 3 each. If they are split 4–2 or 5–1, the algorithm will still produce two clusters, but the recall, as computed by the $\hbox {B}^3$ algorithm, will (slightly) increase with the size of the largest cluster. This has to be kept in mind when comparing naive algorithm performance between data sets with highly similar homography and variability.

We consider the performance of the naive algorithm on each data set as a rough approximation only, and argue that unreasonably good performance (in terms of $\hbox {B}^3$ F1 score as computed by the CONLL scorer) makes a data set less challenging (and thus less useful) as an AND test collection, while average or poor performance is a sign of desirable inherent difficulty. Note that the F1 score is the harmonic mean of precision and recall, which means that two data sets in our experiments can yield similar F1 scores, while one favors precision and the other favors recall.

The naive baseline experiments were performed as follows: For each data set, a CONLL scorer **key** file was created which encoded the correct disambiguation information of the *identified* authorship records in the data set. Unidentified records were left out of the key file, because no correct, gold-standard information is available for them. Then, the naive algorithm was applied to all *identified* authorship records, ignoring the correct identifier information, and using the name information for clustering only. Excluding the unidentified records from disambiguation is important, because failure to do so would cause spurious entries in the disambiguation CONLL scorer **response** file, rendering the disambiguation precision score uninterpretable. When interpreting the results, especially when comparing those of the reviewed data sets with those of the SCAD-zbMATH data set, it is important to keep in mind that the above distinction between *identified* and *unidentified* records does not exist for SCAD-zbMATH, because the latter is a publication-centric, i.e., fully disambiguated data set. This is an important difference which has a significant effect on the naive baseline results. The results for all data sets are given in Table 16, along with the author name homography and variability values from the section “Quantitative analysis and comparison”, repeated here for ease of reference. The highest R and P values per line are highlighted in bold and italics.

It can be seen that in five out of seven of the reviewed data sets, recall is the higher of the two scores. In four of these five (Wang-Arnetminer, KISTI-AD-E-01 (Original and Short names), and Qian-DBLP), the expected low author name variability can also be observed (between 0 and 9%). This prevalence of high recall for the naive algorithm again underlines the strong focus that most existing AND data sets put on homography, and the comparative disregard for variability. In the case of Han-DBLP, however, the high recall cannot simply be explained by low author name variability, as the latter value (23%) is actually the highest among all reviewed data sets. As mentioned above, the distribution of name variants among the authorship records of particular authors comes into play here. In addition, we hypothesize that the high recall is also a result of the exceptionally high rate of publications per author for this data set (37% of authors in Han-DBLP have more than ten publications), and of the fact that this data set does not contain any singleton authors.

**Table 16:** **Naive Disambiguation Algorithm Performance** (full data sets)

Data set	Names	% Homogr.	% Variab.	$\hbox {B}^3\hbox { R}$	$\hbox {B}^3\hbox { P}$	$\hbox {B}^3\hbox { F1}$
Han-DBLP	Mixed	14%	23%	***90***.***12***	23.34	37.08
Culotta-REXA	Mixed	16%	16%	48.03	***88***.***44***	62.26
Wang-Arnetminer	Mixed	80%	01%	***99***.***38***	47.11	63.92
KISTI-AD-E-01	Original	15%	09%	***96***.***00***	90.94	93.40
	Short	88%	00%	***100***.***00***	43.01	60.15
Cota-BDBComp	Mixed	04%	18%	76.10	***94***.***97***	84.49
Qian-DBLP	Mixed	30%	04%	***99***.***83***	55.53	71.22
SCAD-zbMATH	Original	13%	26%	60.87	***93***.***25***	73.66
	Short	18%	11%	82.47	***91***.***79***	86.88

The highest overall value in Table 16 is a recall of 100.00 for KISTI-AD-E-01 (Short names). This perfect recall is a direct consequence of the complete lack of author name variability for this data set. The corresponding precision is only 43.01, the second-lowest value for all data sets. The low precision can be explained by the rather high author name homography of 88%. The F1 score for this data set is 60.15, again the second lowest for all data sets. A similar constellation (low author name variability, very high naive baseline recall, and medium precision) can be observed for Wang-Arnetminer and Qian-DBLP. For all three, the F1 score is sufficiently low for a naive baseline algorithm (between 60.15 and 71.22), if one accepts that the difficulty posed by these data sets lies almost exclusively in name *homography* detection.

On KISTI-AD-E-01 (Original names), on the other hand, the naive algorithm reaches the highest F1 score of as much as 93.40. The pertaining recall and precision scores are both above 90.00.

Among the six reviewed data sets, Culotta-REXA and Cota-BDBComp are the only ones for which the naive baseline algorithm favors precision over recall. Cota-BDBComp has the highest precision of all reviewed data sets, and this again corresponds to a very low author name homography value (actually the lowest of all six reviewed data sets). The relatively high recall of 76.10, however, is less easily explained, as Cota-BDBComp also has the second-highest author name variability (after Han-DBLP). Here, the high percentage of singleton authors (74%) provides an explanation, because singleton authors with unique names form trivially correct one-author clusters for the $\hbox {B}^3$ evaluation. The resulting F1 score for Cota-BDBComp is the second-highest for all reviewed data sets. The homography and variability values for the Culotta-REXA data set bear some similarity to those of SCAD-zbMATH (Short names), although their naive algorithm recall is considerably different: We hypothesize that the lower recall for Culotta-REXA can again be seen as an effect of the higher percentage of singleton authors (66%) in comparison to SCAD-zbMATH (Short names) (41%), in combination with the much smaller size of the former. The full evaluation of this hypothesis, however, requires additional qualitative analysis which is beyond the scope of the present paper. Note in passing that Culotta-REXA and Wang-Arnetminer are instances of data sets which yield almost identical F1 scores for the naive algorithm, although one favors precision and the other recall.

In general, given the fact that considerable efforts were undertaken in order to make the six reviewed data sets useful as test collections for AND, the performance of the naive algorithm is surprisingly high. This observation is in line with other results involving simple AND algorithms: Milojević, while being mainly interested in the algorithm itself, and not in data set creation, also found that simple, name-based methods for AND perform very good in the vast majority of cases (Milojević 2013). The good performance of the naive algorithm is even more remarkable because in the reviewed data sets, only explicitly selected, ambiguous authorship records are identified, while the majority of simple, unambiguous author names cannot exert a positive effect on disambiguation.

For comparison, the results for the SCAD-zbMATH data set are given at the bottom of Table 16. As already mentioned, the fact that this data set is fully disambiguated has a significant effect on the disambiguation process. Since *all* identified authorship records will be disambiguated by default, the naive algorithm cannot distinguish between difficult, actively selected authors, and authors that are trivial to disambiguate. The effect of this can be observed in the rather good performance: The precision of the naive algorithm is very high (in the low 90 s) for both the original and the short name variant of the data set, and exceeded only by that of Cota-BDBComp. However, the creation of the SCAD-zbMATH data set was optimized for author name variability, and the effect of this optimization is visible in the author name variability values and, more importantly, in the comparably low naive baseline algorithm recall: When the original author names are used, the recall is only 60.87%, the second-lowest of all data sets. When only short names are used, it is considerably higher, but still lower than that of most other data sets. The resulting F1 score when original names are used, while rather high with 73.66, lies between that of the other two data sets that also show a preference for naive baseline algorithm precision: Culotta-REXA has an F1 score of 62.26, and overall the lowest recall (48.03) of all data sets. While this also makes it a useful resource for author name variability, Culotta-REXA suffers from some minor issues, and is only about 10% the size of SCAD-zbMATH. Cota-BDBComp, on the other hand, has a slightly higher F1 score of 84.49, which is almost entirely due to its higher recall of 76.10. This, together with the extremely small size of only about 1% of SCAD-zbMATH, makes it compare unfavourably to the latter. Therefore, even when used to disambiguate all authorship records without distinction, the SCAD-zbMATH data set turns out to be a useful addition to the existing body of AND data sets.

However, we wanted to find a way to increase the usefulness and versatility of the data set beyond the simple *all-or-nothing* approach. In particular, we wanted to compensate the distorting effect of simple disambiguation cases, without giving up the diagnostic possibilities provided by full disambiguation. The rather simple solution to achieve this is by **selectively disambiguating** only arbitrary sub sets of the full data set. As described above, in the CONLL-based evaluation used here, authorship records can be selectively excluded from disambiguation by removing their author identifiers. While details may vary in other technical setups, e.g., when other evaluation schemes are used, the basic steps remain the same, making the proposed selective disambiguation procedure generally applicable as long as the employed data set is fully disambiguated.

In order to demonstrate the procedure, we created four sub sets^45^ of the full SCAD-zbMATH data set: The first sub set covered only authors with highly variable names. For this, we first determined the ids of all authors with eight or more original name variants (93 ids in total), and then extracted only those publications from the full data set that were authored by at least one of these authors. Then, we temporarily removed the ids of all *other* authors from the extracted publications, and submitted them to the baseline algorithm. A second sub set covered only highly ambiguous author names. We identified all original names that were used by three or more authors (97 names in total), and proceeded exactly as with the previous data set. A third sub set contained all merged publications with authors matched by any of the two previous lists. Finally, a fourth sub set was created that contained only publications by authors named “Simon, L.” and “Tanaka, K.”. All experiments were run using the original author name information. The results can be found in Table 17.^46^

**Table 17:** **Naive Disambiguation Algorithm Performance** (Data Sub Sets)

Data set SCAD-zbMATH	# Ident. Records	% Homogr.	% Variab.	$\hbox {B}^3\hbox { R}$	$\hbox {B}^3\hbox { P}$	$\hbox {B}^3\hbox { F1}$
Top Variable Authors	8.587	00%	100%	42.39	***100***.***00***	59.54
Top Ambiguous Names	1.578	100%	$<1\%$	***99***.***72***	53.82	69.91
Merged	10.162	10%	22%	51.29	***92***.***83***	66.07
“Simon, L.”, “Tanaka, K.”	37	100%	00%	***100***.***00***	21.85	35.86

The results show that the selective disambiguation can effectively bias a given, fully disambiguated data set in several interesting ways. The most obvious effect of introducing this bias is the considerable drop in performance of the naive disambiguation algorithm, which is due to the fact that the positive contribution of simple, unambiguous author names is eliminated.

The first two sub sets display extreme variability and homography. The first sub set (Top Variable Authors), in particular, is unique in that it exhibits maximal author name variability. Data sets created in this way are useful resources for the development and evaluation of blocking methods. Judging from the result of the naive disambiguation algorithm, the third sub set (Merged) is similar to the Culotta-REXA data set. The crucial difference is that in Culotta-REXA, only a fixed, hard-coded number of authorship records are disambiguated and accessible for evaluation, such that no qualified co-author information is available. For the Merged sub set, in contrast, full and qualified co-author information is available in the background, in the full SCAD-zbMATH data set. This way, it is possible to perform and accurately evaluate co-author detection,^47^ to correctly quantify the impact of co-author homography and variability, and to perform error-analysis. The fourth row in Table 17, finally, demonstrates that selective disambiguation can also be used to reduce a publication-centric data set to the point where it reproduces the common, group-based disambiguation schemes described in the section “Discussion”. Here, the naive algorithm fails. It is important to note that the proposed selective disambiguation strategy does not contradict or undermine the requirement that a data set should be publication-centric: While the selected, focussed sub set is author-centric, it is still based on, and has access to, the complete, publication-centric data set.

## Conclusion

This paper provides what we think is the first comprehensive and detailed analysis of data sets for Author Name Disambiguation. The main results of our study are that we found that existing data sets, by way of how they are created, represented, and used, are suitable for the AND tasks that deal with distinguishing authors with homographic names, rather than for the related tasks of handling name variants and blocking.

Also, due to their focus on highly ambiguous names, all of the data sets in the review are author-centric, which means that they systematically ignore a particular author’s co-authors for any given publication. In methodological terms, our review found this to be the most severe drawback: Co-author names can themselves be ambiguous, and co-authors can appear under various names. This is a problem of existing AND algorithms that, due to the lack of publication-centric, i.e., fully disambiguated data sets, could not be adapted to this problem yet. Finally, the data sets in the review were also found to favor publications with many authors, making them more easily accessible to co-author-based methods.

Coming from the background of practical AND, as carried out at DBLP and zbMATH, it was our intention to complement the existing data sets with a new one with properties that would be different in such a way as to challenge the AND state of the art, and thus to promote the development of new AND algorithms and methods. The creation of the data set SCAD-zbMATH took advantage of the fact that we had access to high-quality, manually curated bibliographic data from DBLP and zbMATH. Using a doubly disambiguated portion of this data, we could empirically ensure the high quality of the manual disambiguation, which in turn allowed us to create the new data set at no extra disambiguation or annotation effort. SCAD-zbMATH is the first AND data set to provide, in sufficient quantity, high-quality bibliographical data with properties that are not found in any other comparable resource. These properties include full author name disambiguation, strong coverage of highly variable name spellings, and, thanks to zbMATH identifiers in the data set, systematic access to publication meta data like abstracts, key words, and MSC codes.

We think that our new data set will turn out to be a valuable resource for the AND research community, as it will allow and require the development of methods that tackle facets of AND which have long been neglected. We think that the SCAD-zbMATH data set is more demanding than existing data sets mainly for the following reasons: Given the prevalence of rich co-author information in existing data sets, and the fact that this information is exploited by many AND systems, we expect to see a decrease in performance if the same systems are applied to the SCAD-zbMATH data set with its much sparser co-author information. We expect this negative effect to be even stronger when combined with the selective disambiguation procedure described in this paper, as this will remove the positive influence of simple, unambiguous cases. Also, given the considerable name variability in SCAD-zbMATH and the absence of pre-defined same-name-groups, existing AND systems must improve their blocking strategies, or add some blocking strategy in the first place, to maintain, or establish, efficiency of the disambiguation process.

We also think that our design principles for AND data sets should be observed in the creation of future data sets. This is true in particular for the aspect of full disambiguation: While the SCAD-zbMATH data set is the first fully disambiguated AND data set, full disambiguation is even more important in fields of publication with a higher number of authors per publication. It might also be feasible to retrofit some of the existing data sets from our review by adding full disambiguation information. Then, using the selective disambiguation procedure, original experiments with these data sets can be reproduced, but they can benefit from improved information for error analysis and diagnostics.

The next step now is the application of existing, state-of-the-art AND systems to the SCAD-zbMATH data set in order to examine in how far reported results achieved by these systems are due to the idiosyncrasies of the employed data sets, and to see if the results can be reproduced with the new data set. Detailed analyses of the observed errors will then be the basis for the design and implementation of new AND systems that are better equipped to handle this data, thus pushing the performance of computational AND systems further towards practical application in productive environments.

## Appendix

See Table 18.

**Table 18:** Some quantitative and qualitative properties of AND data sets

Data set	Measure	Total	ID	No ID	Author order	Full + short names	Fully disambiguated	External link	Analysed names
Han-DBLP	Publications	8453			–	–	–	–	–
	Records	27,575	8431	19,144					
	Distinct names	7443	157	7286					
	Distinct authors		479						
Culotta-REXA	Publications	3007			x	–	–	–	x
	Records	9362	3015	6347					
	Distinct names	3411	298	3113					
	Distinct authors		324						
Wang-Arnetminer	Publications	6656			x	–	–	–	–
	Records	23,608	6729	16,879					
	Distinct names	8007	121	7886					
	Distinct authors		1257						
KISTI-AD-E-01 (Full names)	Publications	37613			x	x	–	x	–
	Records	116,565	41,674	74,891					
	Distinct names	35,532	6250	29,282					
	Distinct authors		6921						
KISTI-AD-E-01 (Short names)	Publications	37,613			x	x	–	x	–
	Records	116,565	41,674	74,891					
	Distinct names	22,393	881	21,512					
	Distinct authors		6921						
Cota-BDBComp	Publications	361			–	–	–	–	–
	Records	1251	361	890					
	Distinct names	820	245	575					
	Distinct authors		205						
Qian-DBLP	Publications	6716			–	–	–	x	–
	Records	24,755	6716	18,039					
	Distinct names	8899	672	8227					
	Distinct authors		1200						
SCAD-zbMATH (original names)	Publications	28,321			x	x	x	x	x
	Records	33,810							
	Distinct names	4696							
	Distinct authors	2946							
SCAD-zbMATH (short names)	Publications	28,321			x	x	x	x	x
	Records	33,810							
	Distinct names	2919							
	Distinct authors	2946							

## Footnotes

1 The research described in this paper was conducted in the project *SCAD—Scalable Author Name Disambiguation*, funded in part by the Leibniz Association (Grant SAW-2015-LZI-2), and in part by the Klaus Tschira Foundation.

2 See section “Data set content analysis” for our discussion of these data sets.

3
http://dblp.uni-trier.de/.

4
https://zbmath.org/.

5 Publicly available at https://doi.org/10.5281/zenodo.161333.

6 E.g. a DOI, i.e., *document identifier* (http://www.doi.org).

7 The $\hbox {B}^3$ measure, which is now a widely accepted evaluation measure for AND, has its origins in precisely this task.

8 We prefer the term *homography* over the terms *homonymy* (Esperidião et al. 2014) and *polysemy*: the former includes the aspect of name pronunciation which is irrelevant for written text, and the latter does not apply to proper names because these do not have actual linguistic meaning. Kang et al. (2011) and Shin et al. (2014) use the term *namesake*.

9 Again, for similar reasons, we prefer this term over *synonymy* (Esperidião et al. 2014) or *heteronymous names* (Shin et al. 2014).

10
https://en.wikipedia.org/wiki/Vietnamese_name.

11
http://talk.onevietnam.org/infographic-vietnamese-last-names-how-many-nguyens-are-there-really/.

12
https://zbmath.org/authors/?q=ai:zoladek.henryk.

13
https://zbmath.org/authors/?q=ai:rata.mefodij-f.

14
https://zbmath.org/authors/?q=ai:pukalskyi.ivan-d.

15 In 2015, 80% of the page impressions on the DBLP server were for author pages.

16
http://abel.lis.illinois.edu/author-ity.html.

17 In addition, the Author-ity data would also have been excluded due to what we felt are overly strict licensing conditions.

18 Sometimes also called the *target name* (Kang et al. 2011) or *author in focus*.

19 Cf. sections “Data curation at DBLP” and “Data curation at zbMATH”.

20 Other terms for this include *ambiguous group*, *block*, *name space* (Liu et al. 2014), or *cluster* (Han et al. 2005a, b).

21 Unless otherwise noted, the descriptive names mentioned in the following are not the official data set names. We apply them in this paper for ease of reference.

22
http://clgiles.ist.psu.edu/data/nameset_author-disamb.tar.zip.

23 In general, for the purposes of this review, we did not attempt to correct or reconstruct the original data sets as fully as possible.

24
https://github.com/tapilab/rexa-coref-data.

25 The data set exists in two versions, a simple and a rich one. The latter one contains additional information for some records, including the author’s home page.

26
https://aminer.org/disambiguation.

27 This name is actually used by (Kang et al. 2011).

28
http://www.kisti.re.kr.

29
http://www.lbd.dcc.ufmg.br/lbd/collections/disambiguation/bdbcomp.tar.gz.

30
https://github.com/yaya213/DBLP-Name-Disambiguation-Dataset.

31 The name homography and variability degrees are computed by subtracting the percentage for $n=1$ from 100%.

32 Examples from the data set include “luiz felipe ferreira da silva”, “rafael valle dos santos”, and “antonio mauro barbosa de oliveira”.

33 Cf. sections “Data curation at DBLP” and “Data curation at zbMATH” for a brief description of the blocking process at DBLP and zbMATH.

34 The oldest entry is the very first volume of the famous Crelle’s Journal from 1826.

35
http://msc2010.org.

36 Publication year of the earliest and the most recent publications in zbMATH.

37 The currently supported services are Mathematics Genealogy Project (MGP), wikidata, mathnet.ru, Gemeinsame Normdatei (GND) of the Deutsche Nationalbibliothek, ORCID, arXiv, ResearchGate, and DBLP.

38 This is in contrast to DBLP, where a new profile will be created if no reliable assignment can be performed.

39 Not to be confused with the average number of authors per publication in zbMATH, which also happens to be 1.7.

40 Every request is manually checked by zbMATH’s author identification team prior to execution.

41
http://conll.github.io/reference-coreference-scorers/.

42 Assigning key and response the other way results in the same F1 score, as it only swaps the P and R scores.

43 This implies that other false *MANUAL* records remained uncorrected and could thus, potentially, also enter our data set. Since these cases are very rare, however, we think that the noise they potentially introduce into the data set is acceptable.

44 Publicly available at https://doi.org/10.5281/zenodo.161333.

45 These data sub sets are included with the full SCAD-zbMATH download.

46 The number of authorship records in the Merged row is slightly less than the sum of the two other sub sets, because 3 records were matched by both filters, but were added only once.

47 The naive baseline disambiguation employed here does not make use of co-author information, but the same is true when using actual AND algorithms.
